# Treatment outcomes of severe acute malnutrition in children treated within Outpatient Therapeutic Program (OTP) at Wolaita Zone, Southern Ethiopia: retrospective cross-sectional study

**DOI:** 10.1186/s41043-017-0083-3

**Published:** 2017-03-09

**Authors:** Mulugeta Yohannis Kabalo, Canaan Negash Seifu

**Affiliations:** 1 School of Public Health, Wolaita Sodo University, Southern Ethiopia, P.O.Box 126, Wolaita Sodo, Ethiopia; 2 School of Public Health, Wolaita Sodo University, Southern Ethiopia, P.O.Box 138, Wolaita Sodo, Ethiopia

**Keywords:** Outpatient Therapeutic Program, Severe acute malnutrition, Treatment outcome

## Abstract

**Background:**

Children in third world countries suffer from severe acute malnutrition (SAM) in an extent of public health important. SAM management protocol available this time brought the approach from facility-based to community-based by Outpatient Therapeutic Program (OTP). But, little was known about the treatment outcomes of the program in Ethiopia. Thus, this study was aimed to assess treatment outcomes of SAM and identify factors associated among children treated at OTP in Wolaita Zone.

**Methods:**

A retrospective facility-based cross-sectional study was conducted in OTP records of 794 children, treated at 24 health posts retrieved from January to December 2014. Population proportion to size (PPS) was used to allocate sample for each selected district and OTP sites within district. Individual cards of children were selected by systematic random sampling. Data were entered, thoroughly cleaned, and analyzed in SPSS version 20.

**Results:**

The recovery rate was revealed as 64.9% at 95% CI (61, 68). Death rate, default rate, weight gain, and length of stay were 1.2%, 2.2%, 4.2 g/kg/day, and 6.8 weeks respectively. Children living in <25 min were with 1.53 times higher odds of recovery than children residing in ≥25 min (AOR = 1.53 at 95% CI (1.11, 2.12)). The likelihood of recovery was 2.6 times higher for children with kwashiorkor than for those with marasmus (AOR = 2.62 at 95% CI (1.77, 3.89)). Likewise, children provided with amoxicillin were 1.52 times more likely to recover compared to their counterparts (AOR = 1.52 at 95% CI (1.09, 2.11)).

**Conclusions:**

The recovery rate and weight gain were lower than sphere standard. Distance from OTP, provision of amoxicillin, and type of malnutrition were factors identified as significantly associated with treatment outcome of SAM. Building capacity of OTP service providers and regular monitoring of service provision based on the management protocol were recommended.

## Background

Acute malnutrition is the short-term response to inadequate nutritional intake that often occurs in combination with infections [1]. It is classified as moderate acute malnutrition (MAM) and severe acute malnutrition (SAM) based on their severity [1, 2], explicitly indicated by children weight for height *Z*-score (WHZ) and/or weight for height/length (WFH %), presence of edema, and mid-upper arm circumference (MUAC). Acute malnutrition is defined as SAM when WHZ < −3, MUAC <115 mm, and/or edema +/++ [2]. It contributes to 1.7 million child deaths per year in sub-Saharan Africa and carries nine times higher risk of death than that for a healthy child [1, 3].

The prevalence of SAM in children at developing countries was around 2% [4]. According to the Ethiopian Demographic and Health Survey (EDHS) 2011, 3% of under-five children were severely wasted. The same report in Southern Nations, Nationalities, and Peoples’ Region (SNNPR) indicates the prevalence of severe wasting in under-five children as 1.9% [5]. Moreover, SAM in Wolaita Zone was as about 2.5% from different reports [6, 7]. Thus, it is a public health problem in most developing world including Ethiopia.

Previously, this SAM was managed in health facilities and therapeutic feeding centers (TFC) with its own shortcomings [8, 9]. Some of the challenges were limited coverage and impact, costliness, cross infections, and high mortality rate [4, 10]. To reduce limitations, community-based management of acute malnutrition (CMAM) was endorsed with joint statement of WHO and UNICEF in 2007 [10]. This advances decentralized management system to the community-based approach. Therefore, community outreach, outpatient management of SAM children without medical complications (OTP), inpatient management of SAM with medical complications (SC), and the program address MAM were designed as components of CMAM [2, 4, 9].

OTP serve management of SAM in children aged 6–59 months [4]. Children will be admitted to the program when MUAC <11.5 cm, nutritional edema is +/++, they passed the appetite test, and no medical complications identified [4, 10]. The management of program were mainly with ready-to-use therapeutic foods (RUTF); other routine medications like antibiotics, vitamin A, and folic acid; and dewormings [2, 11]. Children obtain weekly RUTF based on their weight and routine supportive therapies based on their requirement according to SAM management protocol [4, 12].

Children admitted to OTP are discharged by their respective admission criteria [4]. That is, MUAC admittances are assessed for recovery or progress by MUAC and the same works if admitted by WFH%. Nevertheless, admissions with only edema are discharged by anthropometric indicator like MUAC and WFH% as well. Consequently, children were discharged as recovered when MUAC ≥12.5 cm and no edema for at least two consecutive weeks. However, consistently used discharge criteria to declare recovery is 15% weight gain from admission weight for children admitted with wasting and after disappearance of edema for two consecutive weeks for children admitted with edema [10, 13]. Children discharged from OTP are periodically monitored to avoid relapse [14].

The outcomes were declared as recovered, defaulted, died, medical transfer, and non-respondent for treatment, based on the management protocol [4, 10]. The treatment outcomes were compared with international sphere standard requirements to evaluate the program effectiveness [4]. The recovery, death, and default rates were considered as acceptable when >75, <10, and <15% respectively and alarming when <50, >15, and >25% respectively based on international sphere standard [15]. Moreover, weight gain, length of stay, and coverage were thought as acceptable when ≥8 g/kg/day, <4 weeks, and >50–70%, respectively, and considered as alarming when <8 g/kg/day, >6 weeks, and <40% respectively [4, 10, 15].

The performance of OTP for the management of SAM in children was less explored and factor associated with the performance of the program were also not significantly investigated [11, 16]. Since the endorsement of the program, there were little studies done to elucidate the effectiveness of OTP in Ethiopia. Therefore, assessment of treatment outcomes of SAM in children treated at OTP and factors affecting the treatment outcomes require to be studied. Thus, this study fills the information gaps by determining treatment outcomes with SAM management performance indicators and identifying factors associated with treatment outcome among children treated at OTP in Wolaita Zone. That would add significant input for the program to set long-term planning for CMAM services in general and OTP in particular.

## Methods

### Study setting

The study was conducted in Wolaita Zone, SNNPRS, Ethiopia. Wolaita Zone (study area) is one of the zonal administrations in the southern region of Ethiopia, located 390 km south of Addis Ababa. Total population of this study area was estimated as 1,762,682, where 51% were females and 274,978 were children under 5 years of age. The annual report of zonal health office indicated that 19,390 under-five SAM children were managed at OTP in 2014. The study area in general had 12 administrative districts and three town administrations. Likewise, this area share three hospitals, 70 health centers, and 380 health posts [17].

OTP was piloted in Ethiopia, Malawi, Sudan, and Niger in Africa, for its effectiveness on SAM management from 2000–2006 and endorsed in 2007. Thus, piloting was done from 2000 in the study area and the program was legitimated in 2007 [18–21]. Hence, starting from 2007, the program was implemented in the study area. The program had been implemented in all districts of Wolaita Zone, starting from the consent. However, majority of children managed in OTP exist in Boloso Sore, Damot Sore, Damot Gale, and Boloso Bombe districts. Thus, SAM children managed in OTP of Wolaita Zone at the indicated districts were 12,216 (63%) in 2014 [17].

### Study design and period

A retrospective facility-based cross-sectional study was conducted on records from January to December in 2014. The study period of this research work was from July to August in 2015 as of data collection period.

### Source and study population

All children (6–59 months) admitted to OTP of Wolaita Zone, with a diagnosis of SAM in 2014, were source population. All children selected by systematic random sampling from randomly selected OTP sites within selected districts in the study area were study populations.

### Inclusion and exclusion criteria

Records of children admitted to OTP from January to December 2014 were included. Children who are readmitted were excluded. Moreover, children whose socio-demographic variables and admission criteria are not registered in their OTP card were also excluded.

### Sample size determination and sampling procedure

Sample size used for this study was calculated by means of the software Epi-info version 6. The assumptions considered were 95% confidence level, power 80%, and relative risk of appetite test with plumpy‘nut 4.5, from study conducted in Tigray Region, within treatment outcomes of SAM and their determinants in OTP [11]. Thus, the sample size determined was 794 children represented by their medical cards. Population proportion to size (PPS) was used to allocate the required sample size for each woreda and for each OTP site.

**Fig. 1:**
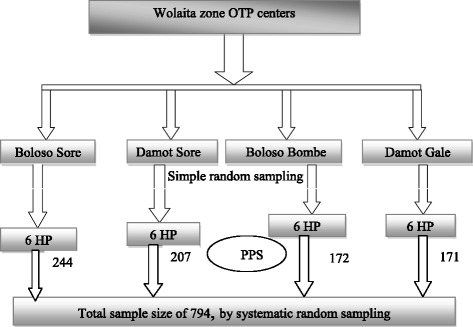
Sampling procedure schematic presentation

There are 12 districts in Wolaita administrative zone. From these districts, four were selected based on their high SAM case flow. The study area in average holds four health centers per district and five satellite health posts per health center in their catchment area. Therefore, in average, there are 20 health posts per district in Wolaita Zone [17]. Of those health posts (HP) or OTP sites, six sites per each district were selected by a simple random sampling technique. Then, a systematic random sampling technique was used to select individual child card (Fig. 1).

### Data collection procedures

Structured and pretested data collection questionnaires were used to collect data from both registration books and children OTP record card. The records were reviewed mainly from children cards, and some variables missing in cards were checked from registration books. Data collectors were those who have 10 + 3 educational level in nursing and are experienced in data collection. Trained data collectors did the record gathering from selected OTP sites and compiled the questionnaire.

### Study variables

*Dependent variable*: treatment outcome

*Independent variables*: socio-demographic: Age, sex, distance to OTP and referral system

Variables related with admission and follow-up: type of malnutrition, physical examination, and history at admission, appetite test by RUTF, length of stay, date of minimum weight gain, RUTF, and other routine medications.

### Operational definitions

*Treatment outcome*: grouped as recovered and not recovered from SAM management at OTP in this study

*Recovered*: children attained 15% weight gain from admission weight for children admitted with wasting and after two consecutive weeks from edema disappeared for edematous children.

*Not recovered*: defined as children discharged from OTP with outcome other than recovery in this study (death, default, non-responder, medical transfer, and falsely recovered).

*Type of malnutrition*: grouped as marasmus (non-edematous), kwashiorkor (edematous), and marasmic kwashiorkor (both edema and severe wasting).

*Distance to OTP site*: indicates time taken from OTP site to house/residence of children by foot in hour/minutes.

*Adequacy of RUTF provision*: sufficiency of RUTF was calculated based on the national SAM management protocol standing from weight of respective children.

### Data quality control and management

Data were collected with close supervision at the time of collection. After proper collection, information were checked up by supervisors for completeness and consistency regularly until data collection is completed. Subsequent to fitting collection, data were carefully entered, cleaned, coded, and analyzed in SPSS version 20.

Systematic data cleaning procedures were carried out to assure data quality of both categorical and continuous variables [22]. Data were cleaned orderly by firstly sorting each variable in ascending order to check for unexpected cases. Variables identified as unexpected and extreme were verified with its respective checklist. Further cleaning was made by selecting randomly 80 (10%) from the total number of participants.

### Data processing and analysis

Data were analyzed by statistical summarization of exposure variables and description of the rates of treatment outcomes. The treatment outcomes were described by recovery, death, default, non-responders for treatment, and medical transfer rates. Both weight gain and length of stay were also calculated based on national protocol [4]. Later, bivariate logistic regression was used to analyze treatment outcome with exposure variables. Multivariable logistic regression was carried out to variables selected by binary regression (*p* value <0.25). Then, predictor variables of treatment outcome were reported by AOR at 95% confidence level (AOR at 95% CI).

## Results

### Socio-demographic characteristics of children

The study included records of 794 children admitted to OTP from January to December 2014 in 24 health posts of four woredas in Wolaita Zone. Of children included, 422 (53.1%) were female and 335 (42.2%) were <24 months of age. The median age at admission was 24 months with IQR of 12 to 36. Children referred to OTP mainly by community volunteers were 386 (65.8%) and came from <25 min of residence from OTP site to home on foot were 378 (53.8%) (Table 1).

**Table 1:** Socio-demographic characteristics of children admitted to OTP in Wolaita Zone in 2014

Variables	Categories of variables	Frequencies	Percentage (%)
Age	<24 months	335	42.2
	≥24 months	459	57.8
Sex	Female	422	53.1
	Male	372	46.9
Referral system	Community volunteers	386	65.8
	HEW	90	15.3
	Self-referred	68	11.6
	Other neighbors	43	7.3
Distance from OTP	≤24 min	378	53.8
	≥25 min	324	46.2

### Nutritional status and co morbidities at admission

Children admitted to OTP based on edema were 273 (34.4%) of the study participants. The rest were admitted based on MUAC. Hence, participants admitted to the program with MUAC <11 cm were 475 (59.8%), 11–11.5 cm were 28 (3.5%), and >11.5 cm were 18(2.3%). Of children with edema at admission, 149 (54.6%), 111 (40.7%), and 13 (4.8%) had grades I, II, and III edema respectively. Moreover, the level of malnutrition was further classified as marasmus 503 (63.4%), kwashiorkor 228 (28.7%), marasmic kwashiorkor 45 (5.7%), and MAM 18 (2.3%) of children included. Children with one or more medical problems were 252 (31.7%) in the study. Of comorbidities, 58 (12.6%) had dehydration, 54 (6.8%) had coughing, and 38 (4.8%) had diarrhea at admission to mention some of the complications (Table 2).

**Table 2:** SAM at admission and medications of children admitted to OTP in Wolaita Zone, 2014

Variables	Categories of variables	Frequencies	Percentage (%)
Type of malnutrition	Marasmus	503	63.4
	Kwashiorkor	228	28.7
	Marasmic kwashiorkor	45	5.7
	MAM	18	2.3
Adequacy of RUTF	Adequate	562	72.1
	Not adequate	217	27.9
Comorbidities	Present	252	31.7
	Absent	542	68.3
Appetite test	Passed	776	97.7
	Failed	18	3.3
Breastfeeding	Yes	296	37.3
	No	468	62.7
Amoxicillin	Medications given	356	44.8
	Not given	438	55.2
Vitamin A	Medications given	203	25.6
	Not given	591	74.4
Folic acid	Medications given	119	15
	Not given	675	85

### RUTF and routine medication provision

RUTF provision is based on admission weight of children. In majority of the study participants, 562 (72.1%) were adequately provided with RUTF in the program. More than half of children adequately supplied were female, 311 (55.3%), and children with admission weight of ≤8 kg (smaller children), 300 (53.4%). Non-edematous children were 372 (66.2%) of participants sufficiently offered RUTF. Routine medications are also provided at OTP, but it was given for some children in the program within the study area. Of children provided medications, 356 (44.8%) were given amoxicillin and 203 (25.6%) were supplied with vitamin A (Table 2).

### Treatment outcomes and international sphere standard references

The performance indicators calculation and further analysis was made for 776 (97.7%) children with SAM. Recovery rate deliberated based on sphere standard was 504 (64.9%) (at 95% CI (61.5, 68.1)) from SAM children who participated in the study. The recovery was within ≤8 weeks for 404 (80.2%) of the recovered children. The rates of death, default, non-responder, medical transfer, and false recovery were 9 (1.2%), 17 (2.2%), 19 (2.4%), 41 (5.3%), and 186 (23.9%), respectively, in this study. The mean (SD) length of stay at OTP was 6.8 (3.2) weeks, and children gain weight in average of 4.2 g/kg/day for length of stay (Table 3).

**Table 3:** Performance indicators of OTP and sphere standard references, Wolaita Zone, 2014

Performance indicators	Frequencies of indicators	International sphere standards references	
		Acceptable	Alarming
Recovery rate	504 (64.9%)	>75%	<50%
Death rate	9 (1.2%)	<10%	>15%
Default rate	17 (2.2%)	<15%	>25%
Average weight gain	4.2 g/kg/days	≥8 g/kg/days	<8 g/kg/days
Length of stay	6.8 weeks	<4 weeks	>6 weeks

### Factors associated with treatment outcome of SAM at OTP

Bivariate analyses were carried out to identify candidate variables for multivariable logistic regression. Variables with *p* value <0.25 at binary logistic regression were selected as candidate for multivariable logistic regression. Thus, variables found associated with treatment outcome at multivariable logistic regression were distance from OTP to home, amoxicillin provision, and type of malnutrition. That is, children residing in <25 min were with 1.53 times higher odds of recovery than those residing in ≥25 min (AOR = 1.53 at 95% CI (1.11, 2.12)). The odds of recovery was 2.6 times higher for children with kwashiorkor than for those with marasmus (AOR = 2.62 at 95% CI (1.77, 3.89)). Children provided with amoxicillin were 1.52 times more likely to recover compared to their counterparts (AOR = 1.52 at 95% CI (1.09, 2.11)) (Table 4).

**Table 4:** Bivariate and multivariable analysis of factors associated with treatment outcome of children in OTP, Wolaita Zone, 2014

Variables (*n*)	Categories	Treatment outcome		OR (95% CI)	AOR (% CI)
		Recovered	Not recovered		
Age (776)	<24 months	213	118	0.95 (0.71,1.29)	
	≥24 months	291	154	1	
Sex (776)	Male	243	119	1.19 (0.89,1.61)	1.17 (0.85,1.62)
	Female	261	153	1	1
Referral system (572)	HEW	66	23	1.66 (0.99,2.77)	
	Self-referred	47	21	1.29 (0.74,2.45)	
	Volunteers	263	152	1	
Distance from OTP (685)	≤24 min	253	116	1.50 (1.10,2.06)*	1.53 (1.11,2.12)*
	≥25 min	187	129	1	1
Type of malnutrition (776)	Kwashiorkor	178	50	2.47 (1.72,3.54)**	2.62 (1.77,3.89)**
	Marasmic kwashiorkor	29	16	1.25 (0.67,2.37)	1.10 (0.53,2.27)
	Marasmus	297	206	1	1
Length of stay (776)	≤6 weeks	245	124	1.13 (0.84,1.52)	
	>6 weeks	259	148	1	
RUTF intake (761)	Adequate	364	184	1.14 (0.82,1.59)	
	Not adequate	135	78	1	
Diarrhea (776)	Absent	486	252	2.14 (1.11,4.12)*	2.13 (0.98,4.61)
	Present	18	20	1	1
Breastfeeding (746)	Yes	188	103	0.97 (0.71,1.32)	
	No	297	158	1	
Amoxicilin provision (776)	Yes	242	111	1.34 (0.99,1.80)	1.52 (1.09,2.11)*
	No	262	161	1	1
Vitamin A intake (776)	Yes	138	60	1.33 (0.94,1.88)	1.41 (0.96,2.05)
	No	366	212	1	1
Date of min.wt. gain (774)	≤3 weeks	446	233	1.22 (0.78,1.89)	
	>3 weeks	58	37	1	

*p < 0.05; **p < 0.01

## Discussion

The finding of this research mainly indicates treatment outcomes of OTP and factors identified as associated with treatment outcome of children treated from SAM. The rates of treatment outcomes were 504 (64.9%), 186 (23.9%), 9 (1.2%), 17 (2.2%), 4.2 g/kg/days, and 6.8 weeks for recovery, false recovery, death, default rate, weight gain, and length of stay respectively. Factors identified as associated with treatment outcome were the distance from OTP to residence by foot, type of malnutrition, and amoxicillin provision in this study. Besides, the incredibly decisive findings this study identified were gaps in reporting recovery rate of SAM in OTP.

Children admitted to the program by edema were 273(34.4%) as specified elsewhere. It was notably large compared to the finding of similar study from Tigray Region (1.6%) [11]. But, the finding was parallel with the report of a related study at Shebedido district (47.4%) [23]. This indicates the admission report by edematous SAM varied by studies. The possible reason might be variation in sampling of children in older age group. That is, majority of children included in this study (57.8%) were aged 2 years and above. Thus, it is credible that the level of edematous SAM is fairly higher at the indicated age group due to stress of stopping breastfeeding.

The result revealed 504 (64.9%) SAM children admitted to OTP were recovered. This indicates the recovery rate was lower than sphere standard acceptable range [4]. The finding was also lower compared to 77.8 and 87% recovery rates from the study done in TFC of Jimma and southern region [8, 24]. However, it was comparable to 61.8 and 67.7% recovery rates from study done in OTP of Tigray Region and Kamba district [11, 16]. The disparities in reports from OTP and TFC might be due to dissimilarity in settings where SAM management was carried out.

Based on the finding, 186 (23.9%) children managed in OTP were discharged having report as “recovered” without attaining discharge criteria of SAM management protocol. Hence, the report in the card of children indicates 690 (88.9%) of SAM children included in the study recovered, while only 64.9% recovered based on the criteria. As per SAM management protocol, children admitted to OTP must be discharged as recovered after attaining 15% weight gain from admission weight for wasted children. Edematous children discharged as cured should be after two successive weeks from edema departure [2, 4, 21]. The likely reason for false recovery reporting might be faulty use of RUTF, since it is sold as a commodity in the study area [25]. Besides, lack of ample awareness on existing SAM management at service providers is also an extra concern.

The recovery rate of SAM at OTP should be >75% to say the program is clinically effective based on sphere standard [15]. But, the rate in this study was lower than the acceptable range of this standard. Beyond the protocol, children managed in the program have failed appetite test 18 (2.3%), marasmic kwashiorkor 45 (5.7%), and grade III edema 13 (1.6%) of partakers. So, the lower recovery rate might be related with admission of children beyond SAM management protocol.

The average weight gain of children admitted with marasmus and recovered was 4.2 g/kg/day being far out of acceptable range of international sphere standard [15]. The finding was slightly lower compared to reports of 5.23 and 5.76 g/kg/day from Tigray Region and Kamba district [11, 16]. The lower average weight gain beneath sphere standard and study findings may be explained by improper utilization of provided therapeutic foods and other supportive medications by children.

The death and default rates were found to be 9 (1.2%) and 17 (2.2%) in this study. Both indicators were in acceptable range of sphere standard [4]. Death rate was comparable with similar study findings from OTP [11, 16, 26]. But, default rate was lesser compared to that from studies in Kamba and Tigray but in line with study from Durame [11, 16, 26]. The relative lesser default rate in this study might be explained by decentralization of the program to health post in the study area.

Children residing in <25 min were 1.53 times more likely to recover than children living in ≥25 min (AOR = 1.53 at 95% CI (1.11, 2.12)). The report offset with the study from Kamba district reported lack of significant association between recovery and residence of children [16]. Other study excluded residence from analysis due to poor registration in the program [11]. The finding of this study can be possibly explained by lack of consistent attendance of children residing in longer distance from OTP. Despite default rate was acceptable, children reside in longer distance were less likely to come OTP site regularly per week than living in near the site.

The odds of recovery was 2.6 times higher for children with kwashiorkor than for children with marasmus (AOR = 2.62 at 95% CI (1.77, 3.89)). That agrees with reports from TFC in Ghana and OTP at sub-Saharan Africa [3, 27]. But, it counters study in TFC in Jimma zone and clinical review of childhood SAM management [28, 29]. Moreover, there is disparity of finding from Kamba district which reported absence of association in type of malnutrition and SAM recovery rate [16]. Children admitted with marasmus had dehydration 35 (60.3%), vomiting 11 (78.6%), and chest retraction 20 (74.1%) in this study. Thus, the possible reason for lower recovery rate in marasmic children might be due to high comorbidities in marasmic children.

Children provided with amoxicillin were 1.52 times more likely to recover compared to their counterparts (AOR = 1.52 at 95% CI (1.77, 3.89)). The finding was in line with the report from OTP in Tigray Region and clinical trial in antibiotics as part of SAM management in India [11, 30]. The likely recovery of children provided with amoxicillin were explained by supportive effect of antibiotics mainly amoxicillin in treatment progress of SAM at OTP. The antibiotic provided for routine treatment must be active against small bowel bacterial overgrowth [4]. Thus, the finding possibly justified that amoxicillin was active against small bowel bacterial overgrowth in SAM children.

### Limitation of the study

Presence of missing information for some variables because of using record and child OTP card as data source would be the possible limitation of this study.

## Conclusions

The recovery rate and weight gain were lower than sphere standard acceptable ranges. On the other hand, death and default rates were within the range of standard in this study. Thus, OTP effectiveness was partial because recovery rate, length of stay, and weight gain were out of sphere standard. Factors identified as significantly associated with treatment outcome of SAM children treated in OTP were distance from OTP site, amoxicillin provision, and type of malnutrition. Moreover, the study leads to conclude as there were gaps in appropriate follow-up of SAM management protocol in the program at the study area. Therefore, stakeholders should manage to shape service providers of OTP with SAM management protocol. To increase the effectiveness of OTP, the performance should be regularly monitored. Special attention was also needed to build capacity of service providers because of current decentralization of OTP to health post. To reveal appropriate utilization of RUTF at home level, further study should be conducted. Besides, coverage of the program also needs to be studied to establish the met need.

## Abbreviations

CMAM: Community-based management of acute malnutrition
NORAD: Norwegian Agency for Development
OTP: Outpatient Therapeutic Program
RUTF: Ready-to-use therapeutic food
SAM: Severe acute malnutrition
